# Proteome Analysis of Spermathecal Fluid and Seminal Plasma Reveals the Mechanism of Sperm Storage in *Amphioctopus Fangsiao*

**DOI:** 10.3390/ani15233495

**Published:** 2025-12-04

**Authors:** Xiaojie Sun, Jiantao Yao, Zexin Huang, Yan Li, Qihao Luo, Weijun Wang, Guohua Sun, Xiaohui Xu, Zan Li, Bin Li, Yanwei Feng, Jianmin Yang

**Affiliations:** 1 Fisheries College, Ludong University, Yantai 264025, China; yw-smile@ldu.edu.cn (X.S.); yjtsober123@163.com (J.Y.); 19163926672@163.com (Z.H.); wwj2530616@163.com (W.W.); sgh_smile@163.com (G.S.); xxh83121@163.com (X.X.); lizanlxm@163.com (Z.L.); 2 Yantai Kongtong Island Industry Co., Ltd., Yantai 264012, China; liyan17853598239@163.com (Y.L.); luoqihao1234@163.com (Q.L.); ytlb1222@163.com (B.L.); 3 Yantai Haiyu Marine Science and Technology Co., Ltd., Yantai 264004, China

**Keywords:** *Amphioctopus fangsiao*, spermathecal fluid, seminal plasma, sperm storage, proteome analysis

## Abstract

The development of males and females of the cephalopod *Amphioctopus fangsiao* is asynchronous. The male produces sperm after maturity for storage in a spermatophore prior to mating. After mating, the sperm enter the female spermatheca for storage until ovulation occurs, a period that lasts for 8 months. This is a biologically uncommon phenomenon because sperm cells generally fail to maintain their ability to fertilize for a long time after being ejaculated. However, the molecular mechanisms of this phenomenon are still not clear. Sperm cells are stored in the male spermatophore and the female spermatheca, each of which provides a suitable environment. To determine the molecular basis of the sperm storage mechanisms in *A. fangsiao*, protein profiles from spermathecal fluid and seminal plasma were characterized separately using mass spectrometry-based proteomics. The antioxidant enzymes superoxide dismutase (SOD), glutathione S-transferase (GST), and Thioredoxin (Trx), and the glycolytic enzymes lactate dehydrogenase (LDH), hexokinase (HK), pyruvate dehydrogenase kinase (PDK), and ATP synthase were significantly enriched in the spermathecal fluid. Catalase (CAT), glyceraldehyde-3-phosphate dehydrogenase (GAPDH), triosephosphate isomerase (TIM), phosphoglycerate kinase (PGK), and Chitinase were significantly enriched in the seminal plasma. The antimicrobial proteins transforming growth factor beta regulator 1 (TBRG1) and interleukin enhancer binding factor 2 (ILF2) and the extracellular matrix-related proteins transforming growth factor beta induced protein (TGFBIp) and thrombospondin type-1 domain-containing protein 4 (THSD4) were also significantly expressed in the spermathecal fluid. These proteins may be crucial for successful long-term sperm storage. We measured the activities of four antioxidant enzymes based on the proteomic results, supporting the antioxidant mechanism during the sperm storage process. This study enhances our understanding of the sperm storage ability of *A. fangsiao*.

## Abstract (plain language summary)

The males and females of the cephalopod *Amphioctopus fangsiao* develop asynchronously. Males reached maturity in September and stored sperm in spermatophores. After mating, sperm enter the female spermatheca for storage until ovulation occurs. This study compared the *A. fangsiao*’s spermathecal fluid (from spermatheca) and seminal plasma (from spermatophore) using proteome, as these two environments support sperm storage. The results found that there were differences in the proteins involved in spermathecal fluid and seminal plasma. Some antioxidant enzymes, glycolytic enzymes, and antimicrobial proteins were identified, which all contribute to sperm storage and thereby ensure the success of fertilization. These findings improve our understanding of *A. fangsiao*’s sperm storage ability.

## 1. Introduction

Reproductive success is a critical factor for sexually reproducing organisms, and to secure this success, various species and distinct sexes have developed highly diverse reproductive strategies, which include mating behaviors, sperm competition, investments in gamete production, blocking of polyspermy, and the provision of parental care [1]. The phenomenon of female sperm storage is a major reproductive strategy related to the reproductive life cycle that has apparently evolved by sexual selection [2]. Sperm storage provides increased fertilization opportunities for species with asynchronous mating and spawning, and the prolonged lifespan of sperm can improve reproductive success [3]. The ability of females to store sperm occurs in numerous taxonomic groups, including mammals, birds, reptiles, amphibians, fish, and insects [4,5,6]. The sperm storage time varies among different species, with durations ranging from a few days to several years. In eusocial insects, sperm can last for as long as decades [5,7,8].

Why do sperm remain fertile for a long time? What mechanisms exist to facilitate sperm storage? Studies have found sperm storage is impacted by various factors, including a low-oxygen environment, changes in metabolism, defenses against oxidative stress, and immune system regulation. In the honey bee *Apis mellifera*, the oxygen content in the female spermatheca is extremely low, and the energy demand of stored sperm relies on anaerobic metabolism, thereby reducing the damage caused by reactive oxygen species (ROS) to sperm [9]. Significant differences in the proteins involved in glycolysis exist between sperm that are ejaculated and stored, with the glycolysis pathway being more prevalent in stored sperm [10]. The expression levels of antioxidant enzymes in the spermatheca of mated honey bee queens are significantly higher than those of virgin queens [11]. In the ant *Crematogaster osakensis*, the low oxygen conditions within the seminal vesicles lead to the stored sperm becoming quiescent, thereby ensuring long-term preservation [12]. In hens, sperm storage tubule cells upregulate transforming growth factor β (TGFB), thus restraining the proliferative activity of lymphocytes and reducing the immune response that may be triggered by the presence of sperm [13].

**Figure 1:**
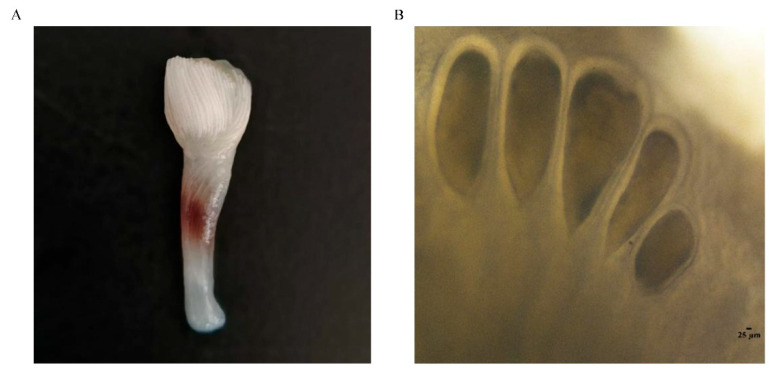
Male spermatophore bundle (**A**) and female spermatheca (**B**) of *Amphioctopus fangsiao*.

In recent years, the fishing volume of cephalopods in China has risen rapidly, indicating their increasing economic importance. The catch volume of cephalopods in 2023 was 593,590 tons, and in 2024, it rose to 597,931 tons [14]. *Amphioctopus fangsiao* (Cephalopoda, Octopoda, Octopodidae) is distributed in the waters of the western Pacific Ocean, and is an important species along the northern coast of China [15,16]. Owing to its high nutritional value and superior flesh quality, it is popular among consumers. The octopus is normally an annual species, with a short life cycle and rapid growth, making it an excellent variety for artificial breeding [17]. The *A. fangsiao* males and females develop asynchronously, with the males’ testes usually reaching maturity in September and females maturing in February, laying eggs in May of the following year. Mature sperm are packaged into spermatophores and stored until mating. The spermatophores are concentrated to form the spermatophore bundles (Figure 1A). Mature males mate with females at the end of September, during which the hectocotylized arm of the male *A. fangsiao* carries the spermatophore to the female mantle cavity, and the sperm pass through the oviduct and are stored within spermathecas (Figure 1B) of the oviduct glands until the female lays eggs after a period of 8 months [18]. Sperm storage in mollusks has been reported in cephalopods [19], and *A. fangsiao,* with eight-month sperm storage, is an ideal species for studying sperm storage in cephalopods. Regarding the sperm storage of *A. fangsiao*, the structural traits and modification of the oviductal glands that are adapted for sperm storage and fertilization have been analyzed in the early stages, and key genes and pathways implicated in sperm storage have been preliminarily identified using transcriptome [20,21]. For the study of sperm storage mechanisms in detail, it is necessary to adopt multi-omics methods from different perspectives.

Data-independent acquisition (DIA) quantitative proteomics has become the technology of choice for the high-throughput characterization of proteins and proteomes. Unlike data-dependent acquisition (DDA), DIA techniques acquire both first mass spectrometry (MS1) and second mass spectrometry (MS2) data without bias to precursor ion selection, overcoming the limitations of inherent irreproducibility and undersampling of DDA [22]. The DIA technology can collect all ion information, achieving a higher data coverage rate, reducing the randomness of data collection, and thereby obtaining very high stability and reproducibility. By using the DIA method for fragment ion quantification, the precision, accuracy and linear range of quantification have been significantly improved [23]. As the specific environment in which sperm are stored in *A. fangsiao* has not been examined in depth, this study conducted DIA proteomic analysis on two environments, the male seminal plasma and the female spermathecal fluid, to identify proteins involved in sperm storage. Based on the proteomic results, the identified antioxidant enzymes were assayed for their activities. This research offers a more in-depth insight into the sperm storage capacity of *A. fangsiao*.

## 2. Materials and Methods

### 2.1. Sample Collection

Male and female *A. fangsiao* specimens, each having a body weight of 43.9 ± 3 g, were sampled from the Yellow Sea waters adjacent to Yantai in December 2023. The sperm storage of *A. fangsiao* occurs from the end of September to May of the following year. December is the middle stage of sperm storage, during which the spermathecal fluid and seminal plasma tend to stabilize. To minimize the impact of environmental changes on their physiological state, the collected individuals were temporarily kept in two dedicated glass tanks (length: 120 cm, width: 60 cm, height: 60 cm) for 3 days, and functional facilities including air stones (to maintain water oxygen levels) and tile pots (to simulate their natural shelter habitats) were arranged in the tanks to promote the octopuses’ smooth acclimatization.

To isolate spermathecal fluid, spermathecae were isolated from the oviduct glands of female *A. fangsiao* using sterilized dissection instruments. The isolated spermathecae were first gently washed three times with precooled phosphate-buffered saline (PBS, pH 7.4) to eliminate potential contamination from adjacent surrounding tissues. A sterilized tungsten needle was used to puncture a small hole in the spermatheca, and a sterilized fine glass capillary tube was inserted to collect the internal spermathecal fluid. The collected fluid was immediately transferred into a centrifuge tube pre-filled with PBS and maintained on ice to preserve its biological activity. All spermathecal fluid of each individual was placed in a single tube; these tubes were then centrifuged to separate sperm and tissue debris from the supernatant. The supernatant was rapidly snap-frozen in liquid nitrogen. For the collection of seminal plasma, the spermatophores were dissected from the male reproductive system and then sectioned after washing with PBS. The seminal plasma was collected using a sterilized fine glass capillary tube. The collected seminal plasma of each individual was released into a tube containing PBS and centrifuged to obtain the supernatant. In order to meet the sample volume requirements for proteomic testing, a total of 12 female *A. fangsiao* were used for sampling, with spermathecal fluid from every four individuals pooled into one sample, and three independent parallel groups established (designated as SF1, SF2, and SF3). For males, a total of 6 *A. fangsiao* were used for sampling, with seminal plasma from every two individuals pooled into one sample, and three independent parallel groups established (designated as SP1, SP2, SP3).

### 2.2. Preparation of Proteomic Samples

Samples were mixed with an appropriate quantity of DB lysis buffer (8 M Urea, 100 mM TEAB, pH 8.5) and centrifuged (12,000× *g* for 15 min at 4 °C), with the resulting supernatant collected for further steps. To the supernatant, 1 M 1,4-Dithiothreitol (DTT) was added, and the mixture was incubated at 56 °C for 1 h to induce protein reduction. Following this, the solution was treated with sufficient iodoacetamide (IAM) for alkylation: the reaction proceeded at room temperature in the dark for 1 h, after which the tube was immersed in an ice bath for 2 min to stop the reaction.

BSA standard protein solution was prepared according to the instructions of the Bradford protein quantitative kit (Beyotime Biotechnology, Shanghai, China), with gradient concentration ranging from 0 to 0.5 g/L. BSA standard protein solutions and sample solutions with different dilution multiples were added into a 96-well plate, respectively, and the volume in each well was 20 µL. Each gradient was repeated three times. The plate was added 180 µL G250 dye solution quickly and placed at room temperature for 5 min. The absorbance at 595 nm was detected. The standard curve was drawn with the absorbance of standard protein solution, and the protein concentration of the sample was calculated. A total of 20 μg of the protein sample was loaded onto 12% SDS-PAGE gel electrophoresis, wherein the concentrated gel was performed at 80 V for 20 min, and the separation gel was performed at 120 V for 90 min. The gel was stained with Coomassie brilliant blue R-250 and decolored until the bands were visualized clearly.

### 2.3. Trypsin Treatment

DB lysis buffer was first used to adjust each protein sample to a standardized volume of 100 μL, and then trypsin, along with 100 mM TEAB buffer, was added sequentially. The mixture was thoroughly mixed and digested at 37 °C for 4 h; additional trypsin and CaCl_2_ were then supplemented, and the sample was allowed to digest overnight. Formic acid was added to the digested sample to adjust the pH to below 3, and the mixture was centrifuged at 12,000× *g* for 5 min at room temperature. The collected supernatant was slowly loaded onto a C18 desalting column, which was washed three times with washing buffer (0.1% formic acid, 3% acetonitrile) before elution buffer (0.1% formic acid, 70% acetonitrile) was added. The eluate from each sample was collected and lyophilized.

### 2.4. LC-MS/MS Analysis-DIA Mode

Mobile Phase A (100% water + 0.1% formic acid) and Mobile Phase B (80% acetonitrile +0.1% formic acid) were prepared first. The lyophilized powder was dissolved in 10 µL of Mobile Phase A, centrifuged at 14,000× *g* for 20 min at 4 °C, and 200 ng of the supernatant was used for LC-MS detection. A Vanquish Neo upgraded UHPLC system was used for separation, with a C18 pre-column (5 mm × 300 μm, 5 μm, Thermo Fisher Scientific, Waltham, MA, USA) and a C18 analytical column (PepMap™ Neo UHPLC, 150 µm × 15 cm, 2 μm, Thermo Fisher Scientific, Waltham, MA, USA)—both columns were heated to 50 °C in a column oven. Mass spectrometry analysis was conducted using a Thermo Orbitrap Astral mass spectrometer (Thermo Fisher Scientific, Waltham, MA, USA) equipped with an Easy-spray (ESI) ion source. The key parameters were set as follows: ion spray voltage = 1.9 kV, ion transfer tube temperature = 290 °C, and the instrument was in DIA mode. For MS1: full scan range = *m*/*z* 380–980, resolution = 240,000 (at 200 *m*/*z*), AGC = 500%, parent ion window size = 2-Th, DIA window number = 300. For MS2: NCE = 25%, sub-ion acquisition range = *m*/*z* 150–2000, sub-ion resolution (Astral) = 80,000, maximal injection time = 3 ms.

### 2.5. Bioinformatic Analysis

Raw mass spectrometry files were searched and analyzed using DIA-NN library search software against the CDS predicted results from the transcriptome assembly conducted by the research group previously [24]. Parameters were set as follows: precursor ion mass tolerance = 10 ppm, fragment ion mass tolerance = 0.02 Da. The immobilizing modification was an alkylating modification of cysteine; the variable modification was a methionine oxidizing modification, and N-terminal modifications were acetylation, loss of methionine, and loss of methionine + acetylation. One missed cleavage site at most was allowed. To enhance result quality, DIA-NN software (version 2.3.0) filtered the search results: only PSMs with ≥99% confidence were retained, along with credible spectral peptides and proteins. A false discovery rate (FDR) validation analysis was then performed, excluding peptides and proteins with FDR > 1%. Principal Component Analysis (PCA) and Coefficient of Variation (CV) analysis were performed on the samples using R-3.4.3 and Python-3.5.0. Differentially expressed proteins (DEPs) were defined as those with *p* < 0.05 and absolute fold change (FC) ≥ 2, as well as those with FC ≤ 0.05 or FC = inf between the two groups. To identify DEPs associated with sperm storage, functional and localization analyses were conducted. Gene Ontology (GO) and InterPro (IPR) functional analysis were conducted using the interproscan program against the non-redundant protein database (including Pfam, PRINTS, ProDom, SMART, ProSite, PANTHER) [25]. The databases of KEGG (Kyoto Encyclopedia of Genes and Genomes) were used to analyze the protein pathway.

### 2.6. Antioxidant Enzyme Activity Assays

In this study, we measured the activities of four antioxidant enzymes between spermathecal fluid and seminal plasma. Superoxide dismutase (SOD) was measured using the hydroxylamine method, glutathione S-transferase (GST) was measured using the colorimetric method, thioredoxin reductase (TrxR) was measured using the colorimetric method, and catalase (CAT) was measured using visible light method. All assays were performed according to the instructions of the corresponding kits, which were purchased from Nanjing Jiancheng Bioengineering Institute (Nanjing, China). Student’s *t*-test was used in SPSS Statistics 27 (IBM, Armonk, NY, USA) to analyze the activity of four enzymes differences between the two groups; results were considered significant when *p* < 0.05. All data were obtained from no fewer than three independent experiments.

## 3. Results

### 3.1. PCA and CV Analysis

**Figure 2:**
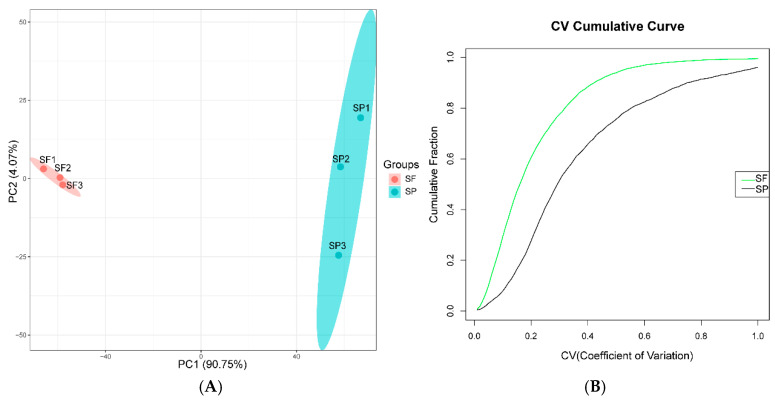
(**A**) PCA of the quantified proteins (red represents the SF group, and green represents the SP group). (**B**) Distribution of CV (green line represents the SF group, and the black line represents the SP group).

Eigenvalues are quantitative indicators of the variance of principal components in PCA, reflecting the contribution of each principal component to the total variation in the data; vectors represent the loading coefficients of protein expression levels on the principal components, indicating the strength of the association between proteins and principal components. In this study, PCA results showed that PC1 accounted for 90.75% of the variance (Figure 2A) and PC2 accounted for 4.07%. PC1 mainly reflects the core differences between the SF and the SP, while PC2 mainly reflects the minor fluctuations within the samples of the group. The two types of samples were completely separated along the PC1 axis, and the three parallel samples within each group were closely clustered, indicating significant differences between sample groups and good reproducibility within groups. CV analysis showed that there is a difference in the distribution of variation between the two groups, with the SF group showing a lower overall level of variation than the SP group (Figure 2B), further validating the stability of the experimental results.

### 3.2. DEPs Between SF and SP

**Figure 3:**
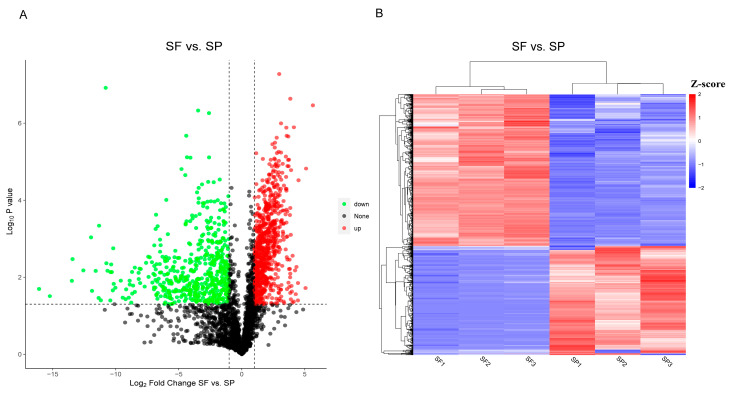
Volcano plot (**A**) (green dots represent downregulated proteins, red dots represent upregulated proteins, and black dots represent proteins with no difference.) and cluster heatmap (**B**) (blue area represent downregulated proteins, red area represent upregulated proteins.) of the DEPs between the SF and SP groups.

This experiment used DIA to establish protein profiles based on spermathecal fluid and seminal plasma samples. A total of 6434 proteins were identified across all samples and used to assess the differences between the two sperm storage environments. We identified a total of 3749 significantly DEPs. Compared to the SP group, 3145 proteins were upregulated, and 604 proteins were downregulated in the SF group. Differential protein abundance distributions were characterized using volcano plots (Figure 3A). The overall similarity of DEPs abundance patterns was illustrated by a hierarchical cluster analysis (Figure 3B). The findings showed that the samples of SF and SP were separated significantly and divided into two main branches. The samples within the groups were in the same cluster, and the consistency was good.

### 3.3. GO Enrichment Analysis

**Figure 4:**
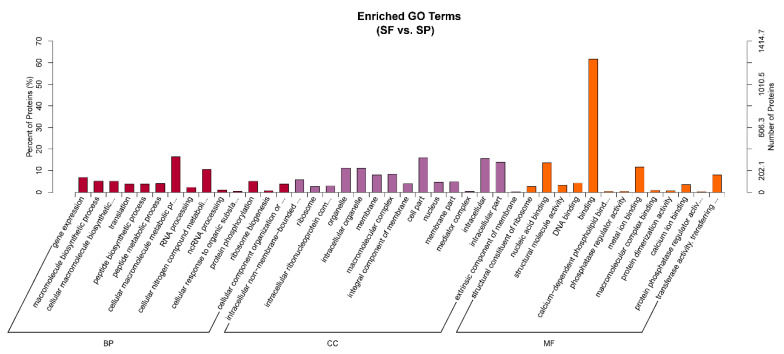
GO functional enrichment analysis of proteins between group SF and group SP (red represents BP, purple represents CC, and orange represents MF). The figure shows the top 42 GO terms enriched by DEPs, along with up to 15 terms in each category.

To explore the potential biological functions of DEPs in the different environments where sperm exist, we initially performed GO enrichment analysis on all DEPs. The DEPs were classified into 3 functional categories: biological process (BP), cellular component (CC), and molecular function (MF) (Figure 4). For the BP category, the main GO terms with significant enrichment included cellular macromolecule metabolic process, cellular nitrogen compound metabolic process, and gene expression. The proteins pyruvate dehydrogenase kinase (PGK) and ATP synthase enriched in the cellular nitrogen compound metabolic process, which is related to glycolysis, may provide an energy supply for sperm storage. In terms of CC, the main enriched GO terms were cell part, intracellular, and intracellular part. For MF, the primary representative GO terms were binding, nucleic acid binding, and metal ion binding. SOD annotated to metal ion binding term is related to the antioxidant mechanism during sperm storage.

### 3.4. KEGG Enrichment Analysis

**Figure 5:**
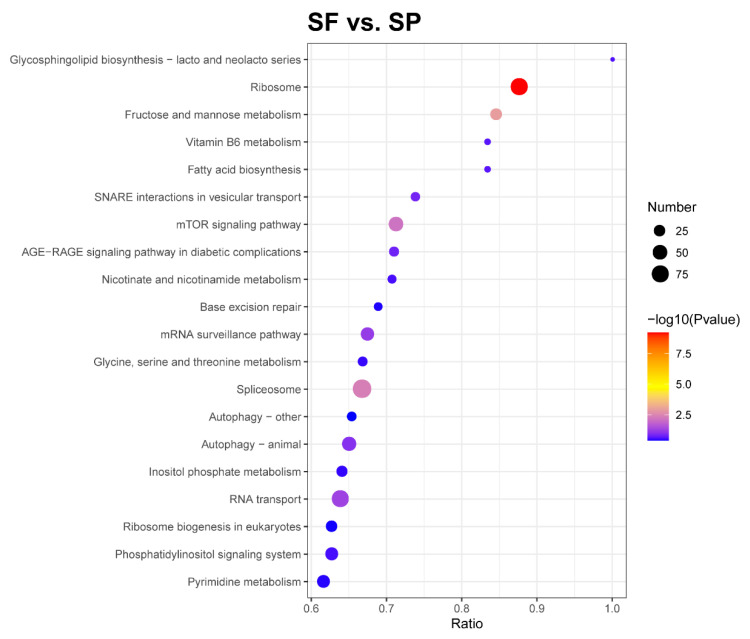
KEGG pathway enrichment of DEPs. The horizontal axis in the graph represents the ratio of the number of DEPs to the total number of proteins identified in the corresponding pathway. The colors of the dots represent the *p*-value from a hypergeometric test. The sizes of the dots represent the number of DEPs in the corresponding pathway.

The KEGG pathway enrichment analysis can identify the most prominent biochemical metabolic pathways and signal transduction pathways in which proteins are involved. The DEPs of the SF and SP groups were distributed among 47 KEGG pathways, and the top 20 enriched pathways are shown in Figure 5. These included ribosome, fructose and mannose metabolism, the spliceosome, the mTOR signaling pathway, RNA transport, the mRNA surveillance pathway, and animal autophagy.

### 3.5. Domain Enrichment Analysis

**Figure 6:**
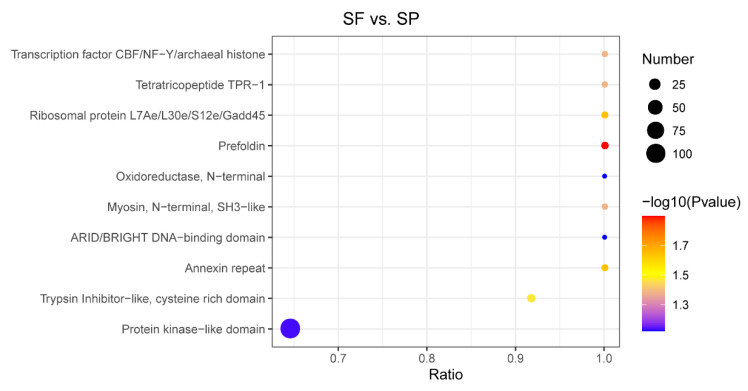
Domain enrichment analysis of DEPs. The horizontal axis represents the ratio of the number of DEPs to the proteins identified in the corresponding domain. The *p*-value is indicated by the color of the dots, while the number of DEPs that possess the corresponding domain is represented by the dot size.

An in-depth study of protein domains serves as one of the key prerequisites for accurately analyzing their biological functions. We performed a domain enrichment analysis of DEPs and characterized the top 10 enriched domains (Figure 6). These included the transcription factor CBF/NF-Y/archaeal histone, tetratricopeptide TPR-1, ribosomal protein L7Ae/L30e/S12e/Gadd45, prefoldin, myosin, N-terminal, SH3-like, Annexin repeat, and Trypsin Inhibitor-like, cysteine-rich domain (*p* < 0.05). Proteins containing these domains will help maintain the integrity of the sperm cell membrane, surface proteins, and internal structure, the potential motility, and act as an immune regulator to protect sperm from pathogens’ invasion.

### 3.6. Subcellular Localization Analysis

**Figure 7:**
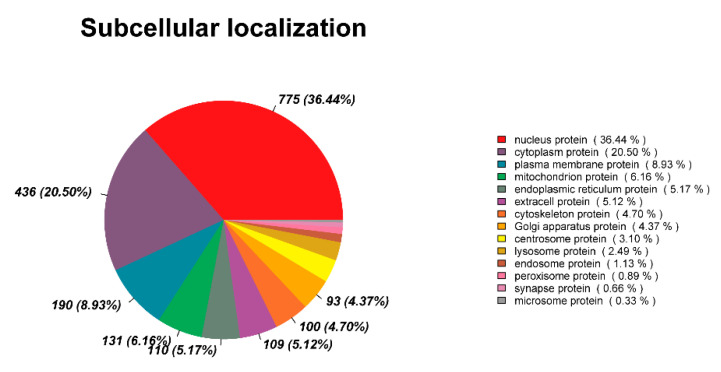
Subcellular localization analysis of DEPs.

Ascertaining the subcellular localization of proteins is crucial for understanding the functions of individual proteins and the organization of the entire cell [26]. The subcellular localization analysis showed nucleus proteins accounted for 36.44% of the DEPs, followed by cytoplasm proteins (20.50%), plasma membrane proteins (8.93%), mitochondrion proteins (6.16%), endoplasmic reticulum proteins (5.17%), and extracellular proteins (5.12%) (Figure 7). Nuclear proteins in spermathecal fluid may play a role in defending against foreign nucleic acids and pathogens, and cytoplasmic proteins may participate in sperm energy supply and oxidative damage repair.

### 3.7. The Critical DEPs Involved in Sperm Storage of A. fangsiao

We identified 16 DEPs that contributed to sperm storage: SOD, GST, Trx, CAT, LDH, HK, PDK, ATP synthase, GAPDH, TIM, PGK, Chitinase, TBRG1, ILF2, TGFBIp, and THSD4 (Table 1). SOD, GST, Trx, LDH, HK, PDK, ATP synthase, TBRG1, ILF2, TGFBIp, and THSD4 were significantly enriched in the spermathecal fluid (group SF, FC ≥ 2.0), while CAT, GAPDH, TIM, PGK, and Chitinase were significantly enriched in the seminal plasma (group SP, FC ≤ 0.05). The proteins SOD, GST, Trx, and CAT are antioxidant enzymes. LDH, HK, PDK, ATP synthase, GAPDH, TIM, and PGK are glycolytic enzymes. There were also antimicrobial-related proteins, such as Chitinase, TBRG1, and ILF2. TGFBIp and THSD4 are extracellular matrix-related proteins.

### 3.8. Catalytic Activity of Antioxidant Enzymes Between SF and SP

**Figure 8:**
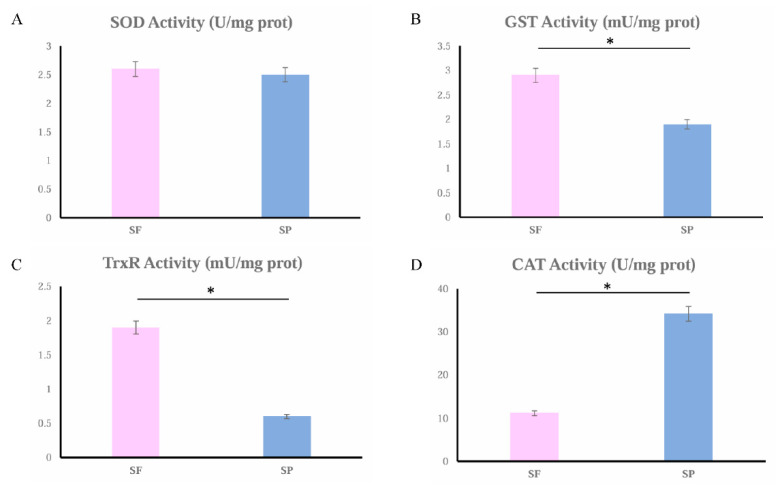
SOD (**A**), GST (**B**), TrxR (**C**), and CAT (**D**) activities in group SF (the pink column chart) and group SP (the blue column chart). Significant differences (*p* < 0.05) are indicated by an asterisk (*).

The catalytic activity of four antioxidant enzymes (SOD, GST, TrxR, and CAT) was measured. These enzymes were not randomly selected; they were screened specifically based on the findings from our proteomic analysis (Table 1). The results are shown in Figure 8. The activities of SOD, GST, and TrxR were higher in the group SF compared with the SP group, and GST and TrxR were significantly different. The enzyme activity of CAT in group SP was significantly higher than that in group SF.

## 4. Discussion

The exceptional sperm storage ability has attracted researchers’ attention, given that sperm storage serves as a crucial reproductive strategy for prolonging sperm viability and thus increasing reproductive success [18]. *A. fangsiao* holds significant economic value as a marine species in the family Octopodidae of Cephalopoda. The sperm of *A. fangsiao* need to be stored in the female spermatheca for 8 months until the eggs are laid. As the spermathecas can be dissected from the oviductal glands, *A. fangsiao* is an ideal model species for studying molluscan sperm storage. Spermathecal fluid and seminal plasma are the environments that contact the stored spermatozoa, and, thus, the present study compared the proteomes of spermathecal fluid and seminal plasma in order to analyze the substances that play a key role in the storage of sperm in these two environments.

### 4.1. Antioxidant Proteins

Many antioxidant proteins were found in both the seminal plasma and spermathecal fluid. Antioxidant proteins are critical for cellular metabolism and essential for cell growth and maintenance, with their main function being to scavenge and degrade ROS [27]. ROS are short-lived, highly reactive molecules formed by the transfer of an electron to an oxygen molecule (O_2_) in aerobic metabolism [28], including both free radicals like the superoxide anion (O_2_•^−^) and hydroxyl radical (•OH), and non-radicals such as hydrogen peroxide (H_2_O_2_). The ROS have a dual role as compounds that are both beneficial and toxic. Low concentrations of ROS have beneficial effects and are involved in immune functions and many cell signaling pathways [29,30]. Studies have found that H_2_O_2_ may participate in capacitation of hamster spermatozoa [31]. In studies on bovine sperm, it was found that H_2_O_2_ concentrations of 10 and 25 micromoles can promote the mature acrosome reactions [32]. However, when ROS levels exceed the detoxification capacity of the cell, oxidative stress may damage membrane lipids, proteins, and local DNA [33]. The sperm cell membrane contains abundant unsaturated lipids, which are major targets for ROS-mediated peroxidation [34,35].

The expression of SOD, GST, and Trx was significantly upregulated, and that of CAT was downregulated in the spermathecal fluid (Table 1). The enzyme activities of SOD, GST, and TrxR were also higher, and that of CAT was lower here. CAT can break down H_2_O_2_ to form water and oxygen molecules [36]. SOD can catalyze the dismutation of superoxide radicals (O_2_•^−^) into hydrogen peroxide (H_2_O_2_) and oxygen (O_2_) [37,38]. H_2_O_2_, a typical ROS endogenously produced during normal metabolism, has been linked to cell dysfunction and disease development when present in excessive amounts [39]. GST is involved in the synthesis and recycling of intracellular Glutathione (GSH) and detoxifies toxic compounds by conjugating them with GSH [40,41,42]. GSH can also indirectly reduce H_2_O_2_ through ascorbic acid [43]. Trx is a mitochondrial antioxidant that, along with TrxR and peroxiredoxin (Prx), scavenges H_2_O_2_ and provides protection against oxidative stress [44]. TrxR is an enzyme that catalyzes the conversion of Trx from its oxidized state to its reduced state by receiving electrons from NADPH [45]. Reduced Trx (Trx-(SH)_2_) transfers electrons to the oxidized Prx (Prx-SOH), restoring it to its reduced form (Prx-SH) and regaining its ability to scavenge H_2_O_2_. SOD, GST/GSH, and TrxR/Trx may work together in the spermathecal fluid, forming a multi-level antioxidant defense network: under conditions of excessive ROS, SOD may convert ROS into H_2_O_2_, then GST/GSH and TrxR/Trx may directly repair the oxidative damage or eliminate H_2_O_2_. However, these antioxidant systems do not completely eliminate ROS. On the one hand, the concentrations of substrates such as GSH and NADPH are dynamic regulatory variables. On the other hand, different antioxidant systems do not function simultaneously. For instance, when SOD, GST, and Trx systems are upregulated, CAT is downregulated. Together, these antioxidant systems may maintain the ROS balance within the spermatheca. This serves a dual purpose: to protect the stored sperm from oxidative damage and contribute to the long-term high-quality storage; to allow ROS to fulfill their essential signaling roles in triggering sperm capacitation and activation during egg-laying. In the seminal plasma, CAT was significantly enriched, and its activity was significantly higher, indicating that CAT plays an important role in antioxidant defense. Sperm produced by males are stored in the spermatophores until mating. The different expression of SOD, GST, Trx, and CAT in the seminal plasma may function to keep ROS levels balanced.

### 4.2. Glycolytic Enzymes

Additional adaptations to limit excessive ROS production include reducing the metabolic rate of stored sperm and/or enhancing their anaerobic metabolism [46]. In studies on the cricket *Gryllus bimaculatus*, it was found that stored sperm exhibited a 37% decrease in metabolic rate and a 42% decrease in ROS production relative to freshly ejaculated sperm. This reduction in metabolic rate may represent a key mechanism for extending sperm longevity within the female storage organs [47]. In the crab *Scylla serrata*, sperm prolonged their storage period through glycolytic metabolism [48,49]. In the quail *Coturnix japonica*, low oxygen levels and high lactate concentrations facilitate the maintenance of sperm in a quiescent state within the sperm storage tubules (SSTs), thereby prolonging sperm viability [50]. The reduced metabolic rate and anaerobic metabolism limit ROS production, which reduces cellular damage from oxidative stress and slows cellular senescence [6,47].

In this study, glycolytic enzymes such as LDH, HK, PDK, and ATP synthase were significantly enriched in the spermathecal fluid (Table 1). LDH is a critical glycolytic enzyme that converts pyruvate to lactate under anaerobic conditions. Its upregulation thereby strengthens glycolytic metabolism and diminishes the cell’s reliance on oxygen under hypoxia [51]. HK initiates glycolysis by phosphorylating glucose to glucose-6-phosphate, and HK also affects apoptotic signaling in the mitochondrial pathway, allowing cells to avoid programmed death [52]. PDK regulates the pyruvate dehydrogenase complex (PDC) activity. The PDC catalyzes the oxidative decarboxylation of pyruvate to generate acetyl-CoA and NADH to participate in the aerobic oxidation of glucose. PDK inhibits PDC activity and downstream energy metabolism by phosphorylating specific serine residues on the PDC E1α subunit, which could cause enhanced glycolysis [53]. Certain cellular stresses, such as hypoxic conditions, may lead to upregulation of ATP synthase, an enzyme that increases ATP production in response to hypoxic stress [54]. The significant enrichment of these glycolytic enzymes suggests that the spermatheca may provide a low-oxygen environment and that the cells need to enhance glycolysis, thereby meeting energy requirements, maintaining normal sperm physiology, and prolonging sperm storage time. Sperm storage in a low-oxygen environment was also found in the ants *Crematogaster osakensis* and *Lasius hayashi* and the honey bee *Apis mellifera* [9,12].

GAPDH, TIM, and PGK were significantly enriched in the seminal plasma (Table 1). GAPDH is a significant enzyme in the glycolytic pathway that catalyzes redox reactions. It reduces NAD^+^ to NADH and converts glyceraldehyde-3-phosphate to 1,3-bisphosphoglycerate [55]. TIM follows fructose bisphosphate aldolase in the glycolytic pathway. Fructose bisphosphate aldolase generates DHAP and d-GAP. TIM catalyzes the further metabolism of DHAP and contributes to the normalization of the glycolytic process [56]. PGK catalyzes the first ATP-generating reaction in the central metabolic pathway of glycolysis, which transforms 1,3-bisphosphoglycerate and ADP into 3-phosphoglycerate and ATP [57]. The significant enrichment of these glycolytic enzymes indicates the presence of glycolytic metabolism in the spermatophores. Spermatophores contain a large number of sperm cells that will be stored until being delivered to the female by the hectocotylized arm at mating time. Glycolysis can provide sufficient energy for sperm in a low oxygen state and, thus, maintain normal physiological functions prior to mating [58].

### 4.3. Antimicrobial Proteins

Bacteria, fungi, and viruses can exert harmful effects on sperm. They may damage sperm DNA, trigger peroxidation of the sperm cell membrane, and impair sperm motility [59,60,61]. There are two primary ways of contact between the microbiota and sperm. Firstly, microorganisms commonly colonize in the male and female reproductive tracts [62,63]. Secondly, when mating occurs, microorganisms from either the external environment or the outer genitals are able to enter the reproductive tract via genital openings and/or wounds on the reproductive organs [64]. To protect sperm from harmful bacteria, the reproductive tissues of both males and females produce a variety of compounds. Throughout the human female reproductive tract (FRT), defensin peptides are expressed, and they exhibit strong antibacterial properties, including the disruption of the membrane and the blockage of bacterial cell wall synthesis [65]. The seminal fluid of the fruit fly *Drosophila melanogaster* contains andropin and drosocin, which have the ability to selectively break down the cell walls of bacteria [66]. Chitinase, which can degrade fungal cell walls, has been detected in the seminal plasma of honey bees and in the spermathecal fluid of both mated and virgin queens [67]. In this study, the contents of TBRG1 and ILF2 were significantly higher in the spermathecal fluid (Table 1). TBRG1 is a growth-inhibitory protein that promotes chromosome stability, and it is also involved in innate immunity in invertebrates [68]. ILF2 regulates viral replication and plays a key role in inhibiting viral infection [69,70]. The upregulation of these proteins could protect the sperm stored in the spermatheca from harmful microbes and facilitate the long-term storage of sperm. Chitinase is significantly enriched in the seminal plasma and may destroy pathogens within spermatophores and, thus, ensure the quality of sperm during mating.

### 4.4. Extracellular Matrix-Related Proteins

The proteomic data demonstrated that TGFBIp and THSD4 were significantly enriched in the spermathecal fluid (Table 1). TGFBIp is a secreted extracellular matrix protein induced by transforming growth factor-beta (TGF-β). It plays a role in the adhesion and migration of a wide range of cells. Effects on adhesion are mediated through interactions with various integrins, which are the only TGFBIp cell surface receptors, and through its function as a linker protein that connects various matrix molecules [71,72]. Depending on this series connection, TGFBIp enhances the adhesion stability of cells in complex matrix environments and lays a “track” for cell migration. The binding of TGFBIp to integrins also acts as a “signal switch” that triggers phosphorylation and activates key intracellular pathways, including protein kinase B (PKB or AKT), extracellular signal-regulated kinase (ERK), focal adhesion kinase (FAK), and paxillin, thus driving cell migration. The upregulation of TGFBIp in the spermathecal fluid in this study may affect the prolonged immobilization of sperm in the spermatheca, and the exit of sperm from the spermatheca at the time of fertilization. THSD4 also belongs to the extracellular matrix-related protein family. In the ant *Lasius japonicus*, THSD4 was significantly enriched in the spermathecal fluid, suggesting that it may provide physical protection by increasing the fluid’s viscosity [73].

In the present study, numerous proteins of unknown function were identified in both the seminal plasma and the spermathecal fluid. In *A. mellifera* and *C. osakensis*, proteins with functions that are yet uncharacterized have also been found in both seminal fluid and the spermathecal fluid [67,73,74,75,76,77]. Therefore, additional study is required to determine whether these proteins of unknown function play a role in sperm storage.

## 5. Conclusions

Long-term sperm storage is a key strategy for species to cope with developmental asynchrony. Exploring the regulatory networks behind it can reveal the evolutionary adaptations of cephalopods in reproductive strategies and fill the gap in the study of the molecular mechanisms of sperm storage in cephalopods. In this study, we performed a proteomic analysis of the spermathecal fluid and seminal plasma in *A. fangsiao*, the two environments in which sperm cells are stored. A total of 3749 differentially expressed proteins were identified. A number of proteins associated with antioxidant functions, glycolysis, antimicrobial defense, cell adhesion, and migration were identified. Antioxidant enzyme activities were also measured. There were differences in the proteins involved in sperm storage between the spermatheca and spermatophore, but all contributed to the storage of sperm, ensuring that subsequent mating and fertilization can proceed. This study revealed the molecular mechanism of long-term sperm storage in *A. fangsiao*, enriched the knowledge of cephalopod reproductive biology, and provided technical support for the artificial breeding of *A. fangsiao*. In the future, we will focus on key regulatory proteins involved in sperm storage, utilizing various technologies, such as protein interaction and gene silencing, to validate their functions and elucidate their regulatory networks.

## Figures and Tables

**Table 1:** Differentially expressed proteins of group SF compared to group SP.

Protein Names	Symbols	FC	*p*-Value
Superoxide dismutase	SOD	2.200	0.0393
Glutathione S-transferase	GST	3.217	0.002
Thioredoxin	Trx	6.220	0.001
Catalase	CAT	0.033	0.012
Lactate dehydrogenase	LDH	2.491	0.011
Hexokinase	HK	Inf	0.000
Pyruvate dehydrogenase kinase	PDK	Inf	0.000
ATP synthase	ATP synthase	Inf	0.000
Glyceraldehyde-3-phosphate dehydrogenase	GAPDH	0.090	0.017
Triosephosphate isomerase	TIM	0.062	0.006
Phosphoglycerate kinase	PGK	0.016	0.003
Transforming growth factor beta regulator 1	TBRG1	Inf	0.000
Interleukin enhancer binding factor 2	ILF2	2.395	0.025
Transforming growth factor beta-induced protein	TGFBIp	7.500	0.040
Thrombospondin type-1 domain-containing protein 4	THSD4	Inf	0.000
Chitinase	Chitinase	0.002	0.046

FC, fold change, a measure used to quantify the differences between proteins between group SF and group SP. If the fold change value is ≥2.0, the expression of the protein in group SF is higher than that in group SP; if the fold change value is ≤0.05, the expression of the protein in group SF is less than that in group SP.

## Data Availability

Data will be made available on request. The mass spectrometry proteomics data have been deposited to the ProteomeXchange Consortium (https://proteomecentral.proteomexchange.org, accessed on 24 November 2025) via the iProX partner repository with the dataset identifier PXD071103.
